# Impact of myo-inositol treatment in women with polycystic ovary syndrome in assisted reproductive technologies

**DOI:** 10.1186/s12978-021-01073-3

**Published:** 2021-01-19

**Authors:** Philippe Merviel, Pandora James, Sarah Bouée, Mathilde Le Guillou, Camille Rince, Charlotte Nachtergaele, Véronique Kerlan

**Affiliations:** 1 Fertility and ART Department, Brest University Hospital, 2 Avenue Foch, 29200 Brest, France; 2 Endocrinology and Metabolism Department, Brest University Hospital, Boulevard Tanguy Prigent, 29200 Brest, France

**Keywords:** Polycystic ovary syndrome (PCOS), Myo-inositol, In vitro fertilization, Intracytoplasmic sperm injection (IVF-ICSI), Embryo quality, Pregnancy

## Abstract

Polycystic ovary syndrome (PCOS) is marked in 30 to 40% by insulin resistance and hyperandrogenism. Myo-inositol (MI) increases insulin sensitivity, decreases hyperandrogenism and improves the menstrual cycle. Its effect during assisted reproductive technologies (ART) has been studied by many authors. We conducted a review of the literature on the impact of MI administration in PCOS women in assisted reproductive technologies. Myo-inositol is effective in normalizing ovarian function, improving oocyte and embryo quality in PCOS, however further evaluations by large multicentre randomized controlled trials are needed to assess the clinical pregnancy and live birth rates in ART.

## Plain English summary

We conducted a review of the literature on the impact of myo-inositol (MI) administration in polycystic ovary syndrome (PCOS) women in assisted reproductive technologies (ART). MI is effective in normalizing ovarian function, improving oocyte and embryo quality in PCOS, however further evaluations by large multicentre randomized controlled trials are needed to assess the clinical pregnancy and live birth rates in ART.

## Introduction

Polycystic ovary syndrome (PCOS) is the most common cause of ovulation disorders, hyperandrogenism and infertility due to ovulatory dysfunction, affecting more than 7% of childbearing age women [1]. PCOS is associated with obesity in 80% of cases and is associated with a metabolic syndrome with insulin resistance in 30–40% of cases, which can aggravate PCOS [2]. Hyperglycemia inhibits hepatic production of Sex Hormone Binding Globulin (SHBG), which leads to an increase of free androgens in the blood circulation, and insulin resistance increases the production of androgens by the theca cells. The management of this insulin resistance is therefore essential in the treatment of PCOS, and is based on nutritional rules, physical activity and other molecules including myo-inositol (MI). The prescription of insulin-sensitizing agents such as metformin is indicated only in cases of glucose intolerance and type 2 diabetes mellitus. In this paper, we aimed to review the role of myo-inositol, a natural insulin sensitizer, on menstrual cycle disorders, ovulation induction and in vitro fertilization/intracytoplasmic sperm injection (IVF/ICSI) outcomes in women with PCOS.

## Mechanism of action of myo-inositol

**Fig. 1:**
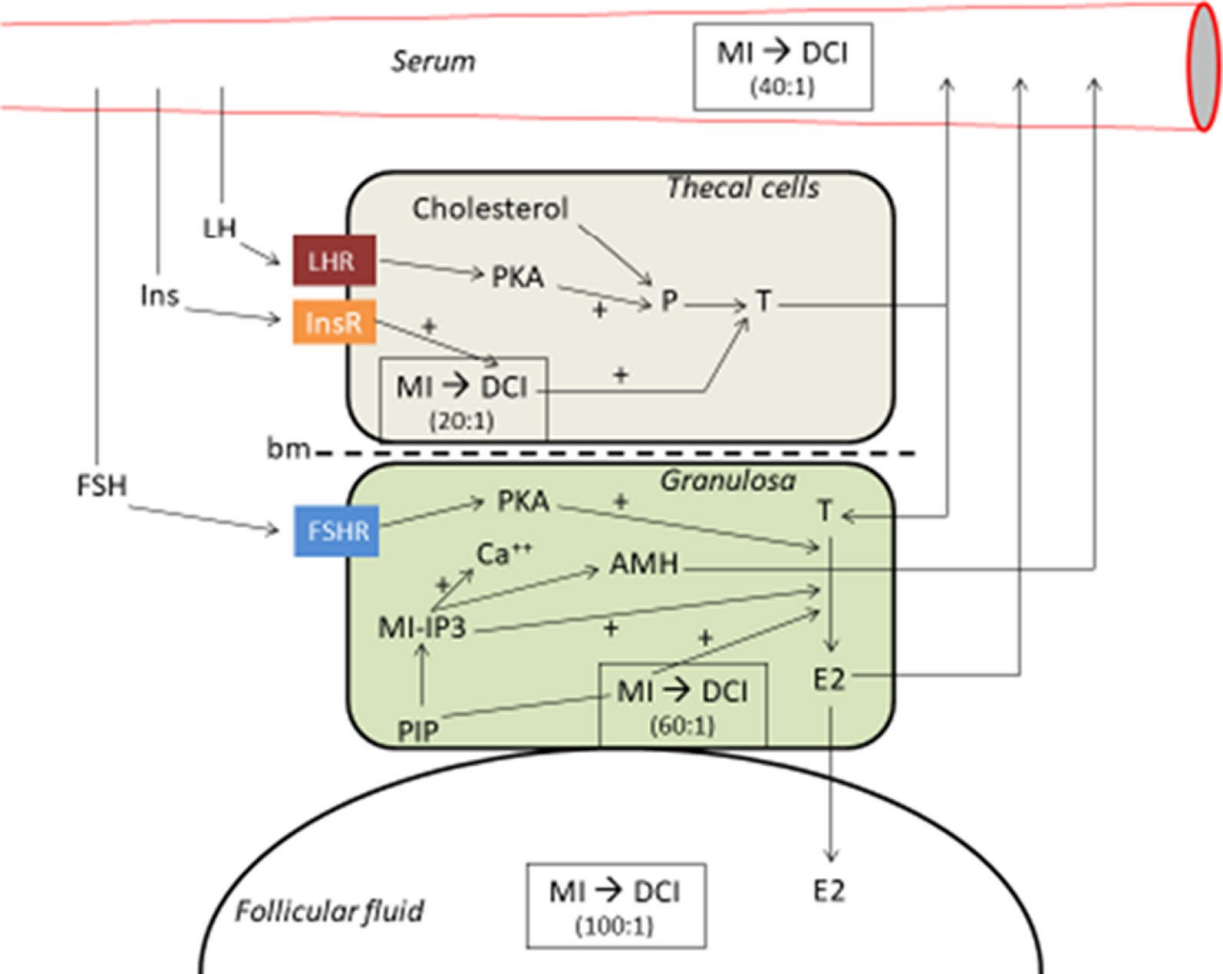
Roles of myo-inositol (MI) in the ovary (original figure from the author, after review of patho-physiologic effects of MI, DCI and others hormones on ovarian cells). MI: myo-inositol; DCI: D-chiro-inositol; (40:1): MI/DCI ratio; LHR: LH receptor; PKA: protein kinase A; P: progesterone; T: testosterone; INs: insulin; InsR: insulin receptor; bm: basalis membrane; PIP: phospho inositide phosphate; IP3: inositide triphosphate; AMH: anti-Müllerian hormone; E2: estradiol; + : stimulating effect

Inositol is a polyalcohol of which there are nine stereoisomers (cyclohexane-1,2,3,4,5,6-hexol). Two of them have been shown to mediate the post-receptor effects of insulin: myo-inositol (MI-cis-1,2,3,5-trans-4,6-cyclohexanehexol) and D-chiro-inositol (DCI-cis-1,2,4-trans-3,5,6-cyclohexanehexol) (DCI). The food categories that contain the highest concentration of inositols are fruits, beans, corn and nuts. DCI negatively interferes with MI absorption at the intestinal level. Uptake of free inositol by tissues occurs by a membrane dependant sodium inositol cotransporter, for which MI has 10 times greater affinity than DCI. MI and DCI are mediated by some inositolphosphoglycans (IPGs), already known as second messengers. These mediators are then internalized and modify enzymatic activity and intracellular metabolism, mimicking the action of insulin. When insulin binds to its receptor, these IPGs are generated by hydrolysis of glycosylphosphatidylinositol (GPI) lipids and/or specific proteins located on the outer part of the cell membrane. Two IPGs are formed: IPG-DCI (or IPG-P) and IPG-MI (or IPG-A). IPG-P will directly activate the glycogen synthase but will also indirectly activate it via the activation of phosphoprotein phosphatase 1 (PP1). IPG-A causes direct glucose uptake and inhibits cAMP protein kinase A and adenylate cyclase, thereby activating PP1. These effects allow a decrease in blood glucose levels (insulin-like effect), regardless of the signal passing through the insulin receptor [3]. In women with PCOS, impaired inositol and/or GPI metabolism contributes to insulin resistance, but obesity plays a specific role in abnormal IPG-P production independently of PCOS [3]. MI decreases body weight, leptin secretion and increases HDL cholesterol [4], but this author have noted that metabolic risk factor benefits of inositol treatment were not observed in the morbidly obese subgroup of women. Thanks to its antioxidant action (SOD, catalase and GSH increase), MI improves cell morphology and growth, as well as the synthesis of lipids participating in cell membranes. Figure 1 summarizes the different actions of MI in the ovary.

Myo-inositol is the most abundant inositol isomer in the human body; DCI is synthesized by an insulin-dependent epimerase that converts MI into DCI. Epimerase activity dysregulation affects MI/DCI ratio, as in PCOS where a defect of MI utilization could impair FSH and insulin signaling. Each organ has a specific MI/DCI ratio related to its function [5]. Therefore, in glycogen storage organs, high levels of DCI have been observed. In the ovary, DCI is responsible for an excess production of insulin-dependent testosterone, whereas MI enhances the action of FSH, via anti-Müllerian hormone (AMH). MI has been found in follicular fluid [2] and appears to improve oocyte and embryo quality. Usually, the MI/DCI ratio is 100:1, whereas in PCOS it is 0.2:1 [6]. When the concentration of MI is reduced in the follicular fluid (which is the case of PCOS, where it is reduced by 500 times), epimerase activation is excessive leading to an excess of DCI, an increase in insulin resistance and an increase in LH levels. If DCI concentrations above the MI/DCI limit ratio of 70:1 in follicular fluid, the blastocyst quality was decreased. The adequate MI/DCI ratio for supplementation is 40:1. This ratio is the best (among seven different ratios between MI and DCI) for PCOS therapy aimed at restoring menstrual cycle and ovulation, increasing progesterone and SHBG and decreasing LH, testosterone and insulin levels [7].

## Effects of MI on menstrual cycle disorders

In PCOS, early follicular growth is excessive, but subsequent progression to a dominant follicle is interrupted (follicular arrest). Intraovarian androgens have been implicated in the excess of follicles and the elevated serum estradiol levels. This increased production of androgens is an inherent property of thecal cells, but it is increased by the surplus of LH and by hyperinsulinism. In women with PCOS, treatment with metformin (MET) ameliorated the insulin sensitivity and decreased the androgens levels, but the limitations to MET use are its gastrointestinal side effects. In this case of PCOS, the place of MI was evaluated. Studies by Zacché et al. [8] and Minozzi et al. [9] show that MI leads to a decrease in LH and androgen levels, as well as a decrease in insulin resistance. Thus, MI is believed to be able to re-establish ovulatory menstrual cycles (especially in obese women with PCOS) but its effect on pregnancy rates is difficult to determine (different diagnoses, insufficient power of studies, non-comparative studies). The second anomaly is the failure to select a dominant follicle, leading to the accumulation of selectable follicles and the typical aspect of polycystic (multifollicular) ovaries when ultrasonography is performed. This phenomenon called follicular arrest is the result of a lack of FSH action and/or premature LH action. Studies have shown the role played by anti-Müllerian hormone (AMH) in inhibiting the follicular response to FSH [1]. Hyperinsulinism, on the other hand, increases the sensitivity of follicles to LH. MI is responsible for a decrease in LH, in the LH/FSH ratio and in testosterone and androstenedione. When ovulation is induced in PCOS women with hyperinsulinism, MI reduces the risk of multifollicular development.

Therefore, MI reduces androgen levels (testosterone and androstenedione), corrects the LH/FSH ratio, restores normal menstrual cycles and induces ovulation, thereby facilitating spontaneous pregnancies by adequate luteal phase progesterone production [10].

## Impact of MI in IVF/ICSI

The role of MI and/or DCI supplementation in women with PCOS undergoing assisted reproductive technologies (ART) to improve oocyte quality, embryo quality and chances to achieve pregnancy has been investigated [11–13]. However, a recent meta-analysis focused on women with PCOS undergoing ICSI found inconclusive evidence on MI efficacy [14]. Table 1 summarizes the main randomized studies on the impact of Myo-inositol (MI), alone or in combination, for induction, ovarian stimulation, or IVF/ICSI in women with PCOS. MI increases the sensitivity of polycystic ovaries to gonadotropins, leading to a reduction in the doses of FSH required:—500 IU for Lagana et al. [15],—327 IU for Zheng et al. [16] (p: 0.007); whereas the opposite is observed for DCI (Carlomagno paradox [17]). MI decreases estradiol levels on the day of ovulation trigger, reduces the number of intermediate-sized follicles and increases the number of large follicles (without increasing the total number of oocytes retrieved), thereby contributing to a reduction of the risk of ovarian hyperstimulation. There is an improvement in oocyte quality and oocyte maturation, an increase in cleavage rate, embryo development (expanded blastocyst) and quality, and an increase in the pregnancy rate in women with PCOS [12]. Chiu et al. [2] demonstrated the link between the concentration of MI in the follicular fluid and oocyte and embryo quality in 53 women with a normal response. He recovered 60 follicular fluids, 38 containing a mature oocyte which was then fertilized (group A) and 22 containing an immature, non-fertilized oocyte (group B). The follicular volume and MI concentration were significantly higher in group A. Significant positive correlations were found between the intrafollicular concentration of MI and the level of estradiol in the follicular fluid, the cleavage rate of fertilized oocytes, the stage of embryos (± 4 cells) and embryo quality (grade). The same author showed that MI supplementation is associated to meiotic progression of mouse germinal vesicle oocytes, enhancing intracellular Ca^2+^ oscillation and leading to the end of meiosis [18]. Sene et al. [19] performed a randomized controlled trial comparing two groups of 25 women with PCOS, one group receiving 4 g of MI + 400 mg of folic acid per day, the other receiving folic acid alone. These pre-treatments started 1 month prior to the start of a GnRH + FSH antagonist protocol, and were continued until the morning of ovulation triggering (via GnRH agonist therapy). The percentage of metaphase II oocytes (78 vs 58%; p: 0.003), the fertilization rate (65 vs 46%; p: 0.03) and the percentage of good quality embryos (p: 0.006) were significantly higher in the MI + folic acid group. While the expression in the granulosa cells of three genes involved in oocyte quality (PGK1, RGS2 and CDC42) was found to be significantly increased in the MI + folic acid group, no significant difference was reported in the concentration of reactive oxygen species (ROS) in the follicular fluid, suggesting that the effect of MI on oocyte quality is independent of its antioxidant action. In this study, no effect was observed concerning the cumulative pregnancy rate (40 vs 35% respectively), contrasting with the study by Artini et al. [20] which found an increase in the pregnancy rate (60 vs 32%; p < 0.05).

**Table 1:** Main randomized studies on the impact of Myo-inositol (MI), alone or in combination, for induction, ovarian stimulation, or IVF/ICSI in women with PCOS

Authors	Material/Methods	Results	Conclusions
Induction/Ovarian stimulation			
Gerli [4]	RCT, 92 women: 47 receiving FA 400 µg/day vs 45 receiving MI 4 g/day et FA 400 µg/day – 3 months	With MI: ovulation (25 vs 15%; p < 0.01), reduced follicular phase duration (24.5 vs 40.5 j; p < 0.05) Clinical pregnancy rates no significantly different	Beneficial effect on ovulation
Raffone [26]	RCT, 120 women: 60 receiving MI 4 g/day et FA 400 µg/day vs 60 receiving 1500 mg/day of MET. If no pregnancy was obtained, addition of rFSH (375 UI/day) during 3 cycles	With MET: 15% ovulated and 18.3% of spontaneous pregnancies. 42 women received rFSH: 26.1% pregnancy rate (cumulative rate 36.6%) With MI: 65% ovulated, 30% of spontaneous pregnancies. 38 women received rFSH, 28.9% pregnancy rate (NS) (cumulative rate: 48.4%—NS)	Myo-inositol appears to be more effective than metformin Bias risks: random sequence generation and incomplete outcome data
Morgante [27]	RCT, 30 women with clomiphene failure, were stimulated with step-down protocol (150 IU FSH – 3 d, then 75 IU/d after): 15 receiving 1.5 g MI (and lactoferrin 100 mg, and bromelain 20 mg)/day vs 15 without MI—1 month before induction	With MI, on the day of hCG: total number of follicles > 15 mm (2.1 vs 3.5) and > 18 mm (1.1 vs 2), estradiol levels 441 vs 955 pg/ml) and cancellation rate (0 vs 40%) were significantly lowerThe clinical pregnancy rate was not significantly different (33.3 vs 13.3%)	MI supplementation increase clinical results in ovulation induction
Ozay [28]	RCT, 196 women: 98 receiving MI 4 g/day and FA 400 µg/day vs 98 receiving FA 400 µg/day – 3 months before FSH and during FSH stimulation and IUI	With MI: 9 spontaneous pregnancies Decrease in total FSH dose and duration of stimulation (p < 0.05); increase in pregnancy rate (18.6 with MI and FA vs 12.2% with FA alone) in IUI	Myo-inositol increases IUI pregnancy rate, and decreases total FSH dose and duration of stimulation Bias risks: allocation concealment and incomplete outcome data
Agrawal [29]	RCT, 120 women: 60 receiving MET 1500 mg/day and MI 1.8 g/day vs 60 receiving MET 1500 mg/day – 3 months If no pregnancy was obtained: FSH and IUI	With MET + MI: improvement in cycle duration Birth rate: 55% (MET + MI) vs 26.6% with MET only (p: 0.002)	Increase in pregnancy rate with MET + MI
IVF/ICSI			
Papaleo [11]	RCT, 60 women without hyperinsulinism: 30 receiving FA 400 µg/day vs 30 receiving MI 4 g/day and FA 400 µg/day, from the start of agonist treatment (long protocol)	With MI: decrease in total dose of rFSH (1958 vs 2383 UI), decrease in stimulation duration (11.4 vs 12.4 j) and decrease in estradiol level on the day of ovulation triggering (2232 vs 2713 pg/ml) (p < 0.05) No difference in the number of oocytes retrieved, but a significant decrease in immature or degenerative oocytes and a tendency for an increase in metaphase II oocytes	Reduction of immature and degenerative oocytes, decrease in estradiol levels on the day of ovulation triggering, no difference in embryo quality and pregnancy rate Bias risks: random sequence generation, allocation concealment and incomplete outcome data
Unfer [6]	RCT, 84 women without hyperinsulinism: 43 undergoing MI 4 g/day vs 41 undergoing DCI 1.2 g/day – 2 month prior to FSH treatment	With MI: increase significantly in mature oocytes (8.21 vs 7.08), good quality embryos (1.64 vs 0.76) and pregnancy rate (51 vs 24%). No difference in the number of oocytes retrieved	Increase in oocyte and embryo quality Increase in the pregnancy rate Bias risks: random sequence generation and incomplete outcome data
Ciotta [30]	RCT, 34 women: 17 undergoing MI 4 g/day and FA 400 µg/day vs 17 undergoing FA 400 µg/day – 3 months prior to FSH treatment and during ovarian stimulation	With MI: increased (p < 0.05) number of oocytes retrieved (12 vs 8.5) and embryos grade 1 (68.1 vs 29%)	Beneficial effect of MI in oocyte maturation Bias risks: random sequence generation, allocation concealment, binding of participants and personnel and incomplete outcome data
Colazingari [12]	RCT, 100 women without hyperinsulinism: 47 undergoing MI 1.1 g/day and DCI 27.6 mg/day vs 53 undergoing DCI 1 g/day – 3 months prior to FSH treatment	With MI + DCI: ≤ 35 years of age: decrease in total FSH dose (1569 vs 1899 IU; p < 0.05) and increase in good quality embryos (0.96 vs 0.73; p < 0.001) With MI + DCI: > 35 years of age: decreased estradiol level on day of hCG trigger and decreased number of oocytes retrieved (p < 0.05), increased number of good quality embryos (p < 0.05)	MI + DCI increases oocyte and embryo quality Bias risks: random sequence generation, allocation concealment, binding of participants and personnel and incomplete outcome data
Pacchiarotti [13]	RCT, 331 women: (A) 165 undergoing MI 4 g/day, FA 400 µg/day and melatonin 3 mg/day vs (B) 166 undergoing MI 4 g/day and FA 400 µg/day vs (control) 195 undergoing FA 400 µg/day – From 1^st^ day of ovarian stimulation to14 days after embryo transfer	Decrease in total FSH dose (A: 2058 IU; B: 3113 and control: 3657; p < 0.001) Increase in the number of metaphase II oocytes (A: 48.2%; B: 35 and control: 38.2) and good quality embryos (A: 45.7%; B: 30.4 and control: 25.6)	MI increases egg and embryo quality Bias risks: random sequence generation and incomplete outcome data
Lesoine [31]	RCT, 29 women: 15 undergoing placebo vs 14 undergoing MI 4 g/day and FA 400 µg/day – 2 months prior to FSH treatment Antagonist protocol	With MI: decrease in follicle/oocyte ratio (p < 0.05), increase (p < 0.05) in fertilization rate and good quality embryos. Decrease (p < 0.05) in the duration of stimulation	Increase in fertilization rate and embryo quality The decrease of the oocyte retrieved can be reduced the risk of hyperstimulation Bias risks: random sequence generation, binding of participants and personnel and incomplete outcome data

The randomized studies were evaluated for the risk of bias using the Cochrane risk of bias assessment tool

RCT: randomized controlled trial; FA: folic acid; MI: myo-insotitol; MET: metformin; IUI: intra-uterine insemination; DCI: D-chiro-inositol; NS: not significant

Two meta-analyses have been published confirming the impact of MI among women with PCOS in IVF/ICSI. Lagana et al. [15] studied the total gonadotropin dose and duration of stimulation in PCOS and non-PCOS women with MI and DCI. Intervention group received 4 g of MI in six studies, 2 g of MI in the Artini' study [20], and 1.1 g MI + 27.6 mg DCI in the Colazingari' study [12]. Comparator was placebo in six studies (400 µg of folic acid in five studies), 1.2 g of DCI in the Unfer' study [6] and 1 g of DCI in the Artini' study [20]. The period of administration was variable, between the day of GnRH agonist administration [11] to 12 [12, 13] or 8 weeks before rFSH administration for Unfer et al. [6]. He reported an effect of MI supplementation on both endpoints in women with PCOS, but only on the total dose in non-PCOS women. The difference is significant between MI and DCI for the decrease in total gonadotropin dose (1953 ± 397 vs. 2360 ± 301 IU, p < 0.01) and duration of stimulation (11.1 ± 0. 8 vs 12.7 ± 1.1 days, p < 0.01), for increased oocyte maturity (8.21 ± 2.39 vs 7.08 ± 2.67 metaphase II oocytes, p < 0.05) and clinical pregnancy rate (22 vs 11%, p < 0.05). No effect is observed on cancelled cycles and on the total number of oocytes retrieved. Zheng et al. [16] conducted a meta-analysis of 6 studies (913 PCOS), showing an increase in clinical pregnancy rates after pre-treatment with MI: 33.3 vs 27.6%, i.e. OR: 1.45 (95% CI 1.04–1.96, p: 0.03). This author reports a 27% decrease in miscarriages (95% CI 0.08–0.50, p: 0.0006). In addition, the proportion of grade 1 embryos is significantly increased, and the number of germinal vesicle, and degenerated oocytes retrieved and the total dose of gonadotropins are significantly reduced. No differences were found in the total number of oocytes, in oocyte maturity, the duration of stimulation or the serum estradiol level on the day of ovulation triggering.

On the contrary, three meta-analyses failed to conclude on the effect of MI in women with PCOS [14, 21, 22]. Mendoza [14] reported, in 8 studies involving 1019 PCOS women, a not significant trend towards improvement in egg quality (OR: 2.2; 95% CI 0.8–5.8), embryo quality (OR 1.6; 95% CI 0.3–6.7) and pregnancy rate (OR: 1.2; 95% CI 0.8–1.8) with MI administration. He concluded that future studies of dose, size and duration of DCI are necessary. Since, two other controlled, randomized, double blind parallel group studies of the same author [21, 22] showed (after 12 weeks of treatment in women with PCOS undergoing ICSI), (i) a significant increase of pregnancy and live birth rates (65.5 vs. 25.9; p: 0.003, and 55.2 vs. 14.8; p: 0.002, respectively) and a decrease of ovarian hyperstimulation syndrome (3.4 vs. 18.5%; p: 0.07) with a 3.6:1 MI/DCI ratio compared to a 40:1 ratio [21]; (ii) a positive influence (p: 0.006) on the quality of the cytoplasm of the oocyte with a 1.8:1 ratio compared to a 20:1 ratio [22]. So, the debate on the appropriate MI/DCI ratio remains unresolved. In the Bhide’ meta-analysis [23] (18 trials included) the primary outcome (changes in anti-Müllerian hormone and antral follicle count before and after treatment), any conclusion was suitable. For the secondary outcomes, no significant differences between MI/DCI and control group were reported on number of oocytes, metaphase II oocytes, top grade embryos, clinical pregnancy rate and risk of ovarian hyperstimulation syndrome. This author noted the very low quality of these studies. In the Cochrane Database analysis [24] (11 trials involving 1472 women with PCOS and IVF), no pooled evidence is available for use of MI versus placebo, insulin-sensitizing and ovulation induction agents for women with PCOS undergoing pre-treatment to IVF. It is unable to show that a myo-inositol treatment increases the chances of pregnant and having a baby, and unclear on whether MI could lower miscarriage rates. In this meta-analysis, the author regretted the small number and the poor quality of the trials, with serious risk of bias associated with poor reporting of methods, imprecision and inconsistency.

Therefore, MI administered 3 months prior to the start of ovarian stimulation, reduces the doses of FSH required for the follicular response, lowers the estradiol level on the day of ovulation triggering, thus reducing the risk of ovarian hyperstimulation [25] and the number of cancelled cycles. Meanwhile, oocyte and embryo quality is increased.

## Conclusions

MI, at a dose of 4 g per day (2 g twice per day), three months prior to ovarian stimulation, is effective in normalizing ovarian function, improving oocyte and embryo quality in PCOS. However, further evaluations by large multicentre randomized controlled trials are needed to assess the clinical pregnancy and live birth rates in ART, because many published studies were heterogeneous. In addition, myo-inositol is a secure and cost-effective alternative in the treatment of PCOS, with no side effects observed in the standard dosage.

## Data Availability

The material contained in this manuscript has not been published, has not been submitted or is not being submitted elsewhere. The datasets used and/or analysed during the current study are available from the corresponding author on reasonable request.

## Abbreviations

PCOS: Polycystic ovarian syndrome
SHBG: Sex-hormone binding protein
MI: Myo-inositol
IVF: In vitro fertilization
ICSI: Intracytoplasmic sperm injection
DCI: D-chiro-inositol
IPG: Inositolphosphoglycan
GPI: Glycosylphosphatidylinositol
PP1: Phosphoprotein phosphatase 1
HDL: High density level
SOD: Superoxide dismutase
GSH: Glutathione
FSH: Folliculostimulating hormone
AMH: Anti-Müllerian hormone
LH: Luteinizing hormone
MET: Metformin
ART: Assisted reproductive technology
Ca^++^: Calcium ion
GnRH: Gonadotropin releasing hormone
ROS: Reactive oxygen species
OR: Odds-ratio
95% CI: Confidence interval at 95
